# No Impact of the Analytical Method Used for Determining Cystatin C on Estimating Glomerular Filtration Rate in Children

**DOI:** 10.3389/fped.2017.00066

**Published:** 2017-04-11

**Authors:** Martin Alberer, Julia Hoefele, Marcus R. Benz, Arend Bökenkamp, Lutz T. Weber

**Affiliations:** ^1^Department of Infectious Diseases and Tropical Medicine, Ludwig-Maximilians University, Munich, Germany; ^2^Institute of Human Genetics, Technical University Munich, Munich, Germany; ^3^Pediatric Nephrology, Children’s and Adolescent’s Hospital, University Hospital of Cologne, Cologne, Germany; ^4^Department of Pediatric Nephrology, VU University Medical Center, Amsterdam, Netherlands

**Keywords:** cystatin C, creatinine, glomerular filtration rate, creatinine clearance, glomerular filtration rate formulas

## Abstract

**Background:**

Measurement of inulin clearance is considered to be the gold standard for determining kidney function in children, but this method is time consuming and expensive. The glomerular filtration rate (GFR) is on the other hand easier to calculate by using various creatinine- and/or cystatin C (Cys C)-based formulas. However, for the determination of serum creatinine (Scr) and Cys C, different and non-interchangeable analytical methods exist. Given the fact that different analytical methods for the determination of creatinine and Cys C were used in order to validate existing GFR formulas, clinicians should be aware of the type used in their local laboratory. In this study, we compared GFR results calculated on the basis of different GFR formulas and either used Scr and Cys C values as determined by the analytical method originally employed for validation or values obtained by an alternative analytical method to evaluate any possible effects on the performance.

**Methods:**

Cys C values determined by means of an immunoturbidimetric assay were used for calculating the GFR using equations in which this analytical method had originally been used for validation. Additionally, these same values were then used in other GFR formulas that had originally been validated using a nephelometric immunoassay for determining Cys C. The effect of using either the compatible or the possibly incompatible analytical method for determining Cys C in the calculation of GFR was assessed in comparison with the GFR measured by creatinine clearance (CrCl).

**Results:**

Unexpectedly, using GFR equations that employed Cys C values derived from a possibly incompatible analytical method did not result in a significant difference concerning the classification of patients as having normal or reduced GFR compared to the classification obtained on the basis of CrCl. Sensitivity and specificity were adequate. On the other hand, formulas using Cys C values derived from a compatible analytical method partly showed insufficient performance when compared to CrCl.

**Conclusion:**

Although clinicians should be aware of applying a GFR formula that is compatible with the locally used analytical method for determining Cys C and creatinine, other factors might be more crucial for the calculation of correct GFR values.

## Introduction

Estimation of the glomerular filtration rate (GFR) is essential for the diagnosis and follow-up of patients with suspected or confirmed kidney disease. Several methods for predicting GFR are available (1). Despite the fact that the inulin clearance test is the gold standard for assessing GFR, it is labor intensive, invasive, and not available in all centers (2). In contrast, methods based on endogenous markers such as serum creatinine (Scr) are convenient and easy to perform (3). Scr concentrations are highly variable depending on muscle mass, activity, nutritional state, and diet and have to be adjusted for gender, body height, and body composition to reflect renal function in pediatric patients accurately. Unlike Scr, serum cystatin C (Cys C) is produced at a constant rate by all nucleated body cells and is independent of age and gender (4–6). Therefore, Cys C might be of special benefit as a marker of GFR in the pediatric population.

Presently, a large array of creatinine- or Cys C-based and combined formulas is available for the calculation of GFR in children. Clinicians may be confused as to which formula would best be used in their setting. Apart from such aspects as the feasibility of the formula and the possibility to use it at the bedside, clinicians should be aware of the various different analytical methods used for determining Scr and Cys C as the type of analytical method employed could influence the performance of the formulas. Scr can be measured by the Jaffe method or by means of enzymatic testing; Cys C, on the other hand, is determined by means of nephelometric or turbidimetric immunoassays. Methods of determining Scr can be used interchangeably with the different GFR formulas if they are isotope dilution mass spectrometry (IDMS) traceable. For Cys C, however, there is no method of adjustment available for the various alternative laboratory tests in routine use so far. Consequently, the choice of a specific GFR formula that uses a Cys C value derived from a possibly incompatible analytical method could lead to incorrect results and to a misclassification of renal function. This study is assessing the performance of different GFR formulas compared to creatinine clearance (CrCl) and evaluates the impact of the type of analytical method used for determining Cys C and Scr on the calculation of the GFR.

## Materials and Methods

### Patients

One hundred forty-one children and adolescents treated between 2004 and 2009 at the Department of Pediatric Nephrology of the Children’s University Hospital, Munich, Germany, were enrolled in this study. The study population consisted of pediatric patients between 2 and 18 years of age with renal disease, metabolic diseases, and malignancies. All pediatric in- and outpatients who were presented to the division of pediatric nephrology for determining of renal function were analyzed. The patients were clinically stable. Patients below the age of 2 were not included, as the children had to have arbitrary voiding for determining the CrCl by 24-h urine collection. Children with gross proteinuria and those on steroids were excluded, as these conditions influence CrCl. Only one set of simultaneous measurements of CrCl, Scr, and Cys C from each patient was used in the analysis. All parameters analyzed were measured on clinical grounds. The data were collected retrospectively and irreversibly anonymized. Therefore, ethical clearance and an informed consent were not required.

### Measurements and Analytical Methods

Patient height and weight were measured in the hospital using standardized scales. Scr (milligrams per deciliter) was determined by an IDMS-traceable method using a Hitachi 911 autoanalyzer. Cys C (mg/l) was measured in serum samples using an immunoturbidimetric assay with a 501c analyzer of the Cobas 6000 series (Roche Diagnostics). The following equations for the calculation of a GFR estimate (eGFR, ml/min/1.73 m^2^) based on Scr, Cys C, or both were tested (Table 1): multivariable Schwartz, creatinine-based Schwartz, Le Bricon, Rule, Filler, and Grubb. While the multiple parameter Schwartz and the Grubb equations were developed using an immunoturbidimetric assay comparable to the assay used in our laboratory, the equations by Le Bricon, Rule, and Filler were established using a nephelometric immunoassay for the measurement of Cys C (4, 7–12).

**Table 1:** **GFR formulas used in the study (4, 7–12)**.

	Creatinine assay	Cys C assay	Equation
**Creatinine- and Cys C-based**			
Multivariable Schwartz	IDMS	Turbidimetry	GFR = 39.1 [height (m)/Scr (mg/dl)]^0.516^ × [1.8/cystatin C (mg/l)]^0.294^ × [30/BUN (mg/dl)]0.169 × 1.099 if male × [height (m)/1.4]0.188
**Creatinine-based**			
Creatinine-based Schwartz	IDMS	–	GFR = K × height (cm)/Scr (mg/dl); K: 0.413
**Cys C-based**			
Grubb	–	Turbidimetry	GFR = 84.69 × [cystatin C (mg/l)]^−1.680^ × 1.384^if <14 years^
Le Bricon	–	Nephelometry	GFR = 78/cystatin C (mg/l) + 4
Rule	–	Nephelometry	GFR = 76.6 × [cystatin C (mg/l)]^−1.16^
Filler	–	Nephelometry	Log (GFR) = 1.962 + [1.123 × log (1/cystatin C (mg/l))]

*GFR, glomerular filtration rate; Cys C, cystatin C; IDMS, isotope dilution mass spectrometry; Scr, serum creatinine; BUN, blood urea nitrogen*.

Creatinine clearance (CrCl) was calculated from Scr and urinary creatinine excretion over 24 h according to the following formula: urine volume (ml) × urine creatinine (mg/dL) × 1.73/serum creatinine (mg/dL) × body surface area (m^2^) × collection time (min). Patients and their parents were instructed on the collection of 24 h urine by dedicated nurses according to standard procedures. Urine was collected as a one-time collection at the time of an outpatient visit or in the course of an inpatient visit. Serum samples were collected on the same day the urine collection was performed.

### Statistical Analysis

The SPSS software (Statistical Package for the Social Sciences, version 20.0, SPSS Inc., Chicago, IL, USA) was used for statistical analysis. Data are presented as mean ± SD (GFR) or median and range [age, height, Scr, Cys C, and blood urea nitrogen (BUN)]. Normal distribution was assessed using the Kolmogorov–Smirnov test. Pearson’s correlation was performed to test for linear correlations between continuous variables. The bias of each eGFR formula compared to CrCl was calculated as the difference between CrCl and the results of the respective eGFR equation. Accuracy was measured as the interquartile range (IQR) of the difference between CrCl and the respective eGFRs and as the proportion of eGFR values within 10, 30, and 50% of measured CrCl. Sensitivity, specificity, positive predictive value (PPV), and negative predictive value (NPV) for the detection of impaired renal function (i.e., CrCl < 90 ml/min/1.73 m^2^) were calculated for each formula with the CrCl serving as gold standard. Bland–Altman plots were created to visualize the performance of the different eGFR formulas. Both receiver operating characteristic (ROC) curves and contingency tables analyzed by McNemar’s test were used to evaluate the correct classification of patient eGFR values into the normal (≥90 ml/min/1.73 m^2^) or pathological (<90 ml/min/1.73 m^2^) range with CrCl serving as gold standard. The level of statistical significance was set at a *p* < 0.05.

## Results

Patient age ranged between 2 and 18 years (median 10.1 years). Sixty-three children (44.7%) were female. Median height was 136.1 cm (range 86.4–191.5 cm) (Table 2). Median Scr was 0.60 mg/dl (range 0.2–11.0 mg/dl), median Cys C 0.78 mg/l (range 0.4–4.6 mg/l), and median BUN 15 mg/dl (range 2–86 mg/dl). Mean CrCl was 116 ± 60 ml/min/1.73 m^2^, 52/141 (37%) patients had a GFR <90 ml/min/1.73 m^2^ as measured by the CrCl (Table 2). The results of the different eGFR equations are summarized in Table 3. All eGFR formulas were highly significantly correlated to CrCl (data not shown).

**Table 2:** **Overview of the demographic data and CKD stages of the patient population (13)**.

Age (median, range)	10.1 (2.4–18.6)
Female (*N*, %)	63 (44.7)
Male (*N*, %)	78 (55.3)
Height in cm (median, range)	136.1 (86.4–191.5)
Weight in kg (median, range)	32.2 (12.4–90.5)
BMI (median, range)	17.3 (12.4–32.0)
CKD stages (*N*, %)	
1	89 (63.1)
2	26 (18.4)
3	21 (14.9)
4	4 (2.8)
5	1 (0.7)

**Table 3:** **Mean glomerular filtration rates estimated by creatinine- and cystatin C (Cys C)-based, creatinine-based, and Cys C-based equations**.

Equation	Value
**Creatinine- and Cys C-based**	
Multivariable Schwartz	90 ± 36 (13–186)
**Creatinine-based**	
Creatinine-based Schwartz	100 ± 48 (4–240)
Creatinine clearance	116 ± 60 (7–369)
**Cys C-based**	
Grubb	161 ± 94 (7–466)
Le Bricon	98 ± 35 (21–181)
Rule	102 ± 40 (13–199)
Filler	114 ± 46 (17–230)

*Values are mean ± SD (range), and all units are ml/min/1.73 m^2^*.

**Figure 1:**
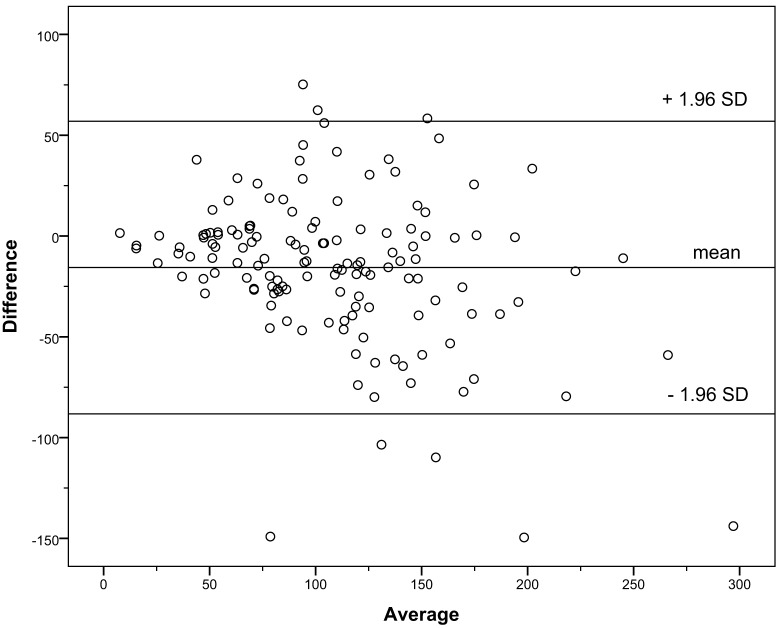
**Bland–Altman plot for the creatinine-based Schwartz formula versus creatinine clearance**.

**Figure 2:**
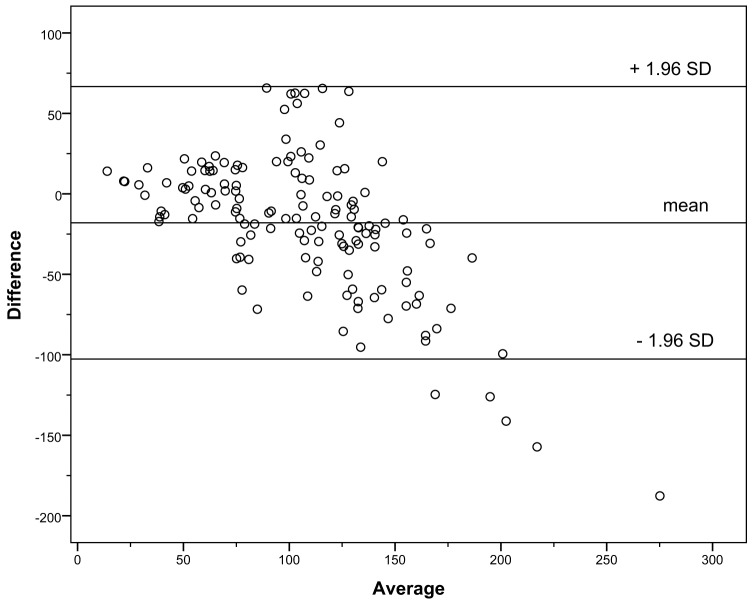
**Bland–Altman plot for the Le Bricon formula versus creatinine clearance**.

Data on bias and precision are presented in Table 4. The multivariable Schwartz, creatinine-based Schwartz, the Le Bricon, and the Rule formulas underestimated the GFR, whereas the other formulas overestimated it. The Filler formula showed only a very small median bias (Table 4). IQRs were large especially for the Grubb formula. Therefore, the proportion of values between ±10, 30, and 50% of CrCl was low for this formula. In the Bland–Altman analysis, the limits of agreement for all formulas were wide, especially in the range of GFR values ≥90 ml/min/1.73 m^2^ but could be substantially reduced if only patients with a GFR of <90 ml/min/1.73 m^2^ were analyzed (data not shown). The Le Bricon, Rule, Filler, and multivariable Schwartz formulas showed a tendency to underestimate the GFR the Grubb formula a tendency to overestimate it with higher values in the Bland–Altman analysis. There was no trend in the case of the creatinine-based Schwartz formula (data shown for the creatinine-based Schwartz formula, Figure 1, and the Le Bricon formula, Figure 2).

**Table 4:** **Median bias, precision (IQR), and accuracy (P10, P30, and P50) for the multivariable Schwartz, creatinine-based Schwartz, Grubb, Le Bricon, Rule, and Filler formulas**.

	Median bias	IQR	P10 (%)	P30 (%)	P50 (%)	Sensitivity (%)	Specificity (%)	PPV (%)	NPV (%)
Multivariable Schwartz	−19.1	43.1	21	64	94	85 (72–93)	73 (64–82)	65 (52–76)	89 (80–95)
Creatinine-based Schwartz	−12.5	30.7	28	67	92	83 (70–92)	76 (66–85)	67 (54–78)	88 (79–95)
Grubb	29.9	93.9	18	41	62	60 (45–73)	89 (80–95)	76 (60–88)	79 (70–87)
Le Bricon	−14.2	46.3	18	60	87	79 (65–89)	82 (73–89)	72 (59–83)	87 (78–93)
Rule	−16.8	42.8	18	62	87	79 (65–89)	78 (67–86)	67 (54–79)	86 (77–93)
Filler	2.6	40.5	26	60	87	73 (59–84)	89 (80–95)	79 (65–90)	95 (76–92)

*IQR, interquartile range; P10, P30, and P50, percentage of eGFR within 10, 30, and 50% of CrCl, respectively. Specificity, sensitivity, positive predictive value (PPV), and negative predictive value (NPV) for all eGFRs*.

**Figure 3:**
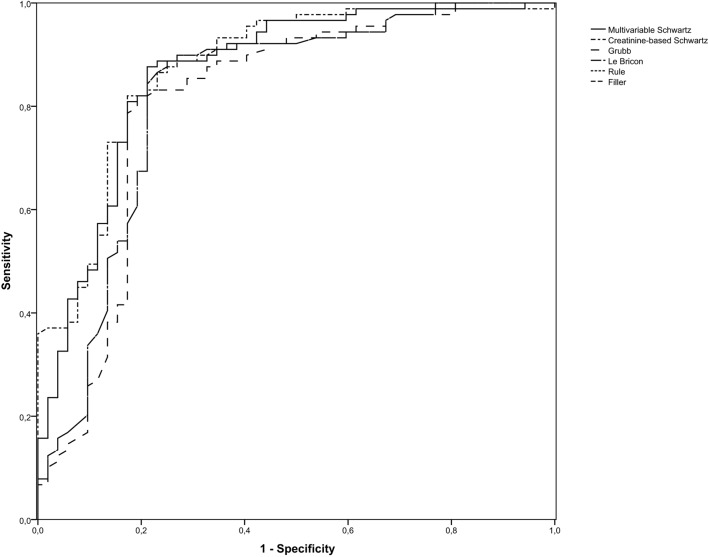
**Receiver operating characteristic curves for the different glomerular filtration rate (GFR) formulas (detection of GFR <90 mL/min/1.73 m^2^)**.

Using the CrCl as gold standard sensitivity, specificity, PPV, and NPV were calculated for the different eGFR formulas (Table 4). The multivariable Schwartz formula and the creatinine-based Schwartz formula showed good sensitivity and specificity compared to CrCl, but the PPV was slightly below 70% in both formulas. The Grubb formula, which is based on the turbidimetric immunoassay for Cys C, had lower sensitivity than the immunonephelometric Filler equation while both had comparable specificity (Table 4). The different eGFR equations were compared with the CrCl in order to ascertain whether they classified patients correctly in the group with normal GFR (≥90 ml/min/1.73 m^2^) or reduced GFR (<90 ml/min/1.73 m^2^). The multivariable Schwartz formula classified 77%, the creatinine-based Schwartz formula 79%, the Grubb formula 78%, the Le Bricon formula 81%, the Rule formula 78%, and the Filler formula 83% of the patients correctly. The multivariable Schwartz, the creatinine-based Schwartz, and the Le Bricon formulas misclassified 14–17% of patients with normal GFR while the Grubb equation incorrectly classified 15% of patients with reduced GFR as normal (Table 5). ROC analysis of the predictive performance of the different formulas (Figure 3) showed comparable areas under the curve ranging from 0.809 to 0.875 (Table 6).

**Table 5:** **Glomerular filtration rate (GFR) estimated by multivariable Schwartz, creatinine-based Schwartz, Grubb, Le Bricon, Rule, and Filler formulas compared to the creatinine clearance (CrCl)**.

	Reduced GFR estimated by CrCl				Normal GFR estimated by CrCl				*p*^a^
	Normal GFR		Reduced GFR		Normal GFR		Reduced GFR		
	*N*	%	*N*	%	*N*	%	*N*	%	
Multivariable Schwartz	8	6	44	31	65	46	24	17	<0.01
Creatinine-based Schwartz	9	6	43	30	68	48	21	15	0.045
Grubb	21	15	31	22	79	56	10	7	0.072
Le Bricon	11	8	41	29	73	52	16	11	0.441
Rule	11	8	41	29	69	49	20	14	0.151
Filler	14	10	38	27	79	56	10	7	0.541

*Reduced estimated GFR defined as <90 ml/min/1.73 m^2^. Normal estimated GFR defined as ≥90 ml/min/1.73 m^2^*.

*^a^McNemar’s test*.

**Table 6:** **Overview of the area under the curve for the different glomerular filtration rate (GFR) formulas (detection of reduced GFR)**.

	Area under the curve
Multivariable Schwartz	0.865*
Creatinine-based Schwartz	0.875*
Grubb	0.809*
Le Bricon	0.821*
Rule	0.821*
Filler	0.821*

*p < 0.001.

## Discussion

Correct estimation of GFR is crucial for detection, evaluation, and treatment of renal diseases (5). The classical parameter to assess renal function is Scr, and GFR is estimated on the basis of creatinine-based formulas. Creatinine-based estimation of GFR, however, has distinct limitations. Recent reports demonstrate that among all available markers Cys C is the best marker of renal function and a valid test for diagnosis of renal impairment (3, 5). Bacchetta et al., for example, reported a good performance of Cys C-based formulas (Le Bricon, Larsson) compared to the inulin clearance (14).

In the present study, the creatinine-based Schwartz formula, the multivariable Schwartz formula, and the cystatin-C-based Grubb, Le Bricon, Rule, and Filler formulas were evaluated in comparison to the CrCl in a cohort of pediatric patients with a variety of renal and extrarenal diseases requiring GFR measurement.

Several pitfalls have to be considered when calculating the GFR by different formulas. Concerning the creatinine-based formulas there are two different ways of measuring Scr values, which are the Jaffe method and the enzymatic assay. In Germany, the Jaffe method is still regarded as standard method for measuring Scr. Although this method is cost-effective, it is hampered by interferences of up to 20% of non-creatinine chromogenes. The problem of interferences has been reduced by the enzymatic assays. Additionally, there is the issue of varying specificities when Scr is measured in different laboratories (15–17). This can lead to an inadequate calculation of the GFR when creatinine-based formulas are used. To overcome this problem, standardization of Scr measurements can be achieved by using a method that is IDMS traceable. Both new Schwartz formulas (creatinine-based and multivariable) have been evaluated using an IDMS-traceable method. Therefore, the Scr measurement at the local hospital has to be IDMS-traceable if these formulas are to be used for calculating the GFR. A similar problem arises when Cys C-based formulas are used. There are two different ways of measuring Cys C: the turbidimetric and the nephelometric immunoassays. Unfortunately, however, the results of these two assays cannot be used interchangeably. To overcome this problem, the IFCC Working Group for the Standardization of Cys C announced in 2010 the availability of the new certified reference material ERM-DA471/IFCC to be used primarily for the calibration of immunoassay-based *in vitro* diagnostic devices concerning the measurement of Cys C (18). In future, the two methods for Cys C measurement could be used interchangeably if this calibrator material is applied. Up to now, it is still necessary to check back with the local laboratory to find out which test method was used.

The formulas that were combined with a compatible analytical method, especially the multivariable Schwartz and the creatinine-based Schwartz formula, showed a good performance in our cohort. Although both Schwartz formulas classified significantly more patients in the group of reduced GFR compared to the CrCl, there is a tendency in the CrCl to overestimate the GFR, which in turn may be responsible for this effect. The Grubb formula showed a lower sensitivity compared to the other formulas, which might lead to insufficient detection of patients with a reduced GFR. On the other hand, the formulas using a value derived from a possibly incompatible analytical method (Le Bricon, Rule, and Filler formulas) showed a comparatively good performance compared to the CrCl. There was no significantly different assessment of the GFR and the percentage of concordant classification was similar to the other adequately applied formulas.

A drawback of our study design is the use of the CrCl as a gold standard. It has to be noted that the CrCl method involves precise urine collection for 24 h, which is hard to obtain in young children and very time consuming (3). Moreover, due to Scr excretion *via* renal tubular secretion, the measurement of CrCl tends to overestimate the true level of GFR (3, 19, 20). This overestimation is difficult to quantify because it can be increased by renal diseases and reduced by certain drugs (20). Therefore, this method is prone to error, and its use as a general standard is limited. In our study, preanalytical mistakes of false sampling were excluded to the greatest possible extent by a sampling protocol that had to be conducted exactly.

As regards the PPV and NPV for detecting a reduced GFR, it has to be pointed out that the prevalence of reduced GFR in our cohort is much higher than in the general population. As PPV and NPV depend on the prevalence, the PPV is expected to be higher and the NPV expected to be lower in our study than if the formulas were to be applied in the general population. Although we did not do a formal sample size calculation, the narrow confidence intervals for sensitivity, specificity, PPV, and NPV show that our study was adequately powered to compare descriptively the eGFR formulas. As there were two significant results in the McNemar’s test, this additionally shows that the sample size used was sufficiently large to detect relevant differences between the CrCl and the eGFR formulas.

In summary, we found no significant impact when using the possibly incompatible Cys C-based eGFR equations. Therefore, clinicians could use their local Cys C values with the Le Bricon, Rule, and Filler eGFR formulas without worrying about an incompatible analytical method. As the method of Scr measurement in our laboratory was IDMS traceable, any impact that an incompatible eGFR equation for Scr might have could not be assessed. This might be of less clinical significance as most laboratories will have established IDMS-traceable methods for evaluating the Scr. Nevertheless, even correctly applied GFR formulas may show insufficient performance when judging the GFR in children. We conclude from our study that using only the compatible analytical method for Cys C when calculating the eGFR is of lesser importance. The imprecision of the formulas used seems to depend on other factors that might be due to the composition of the original cohort to generate the algorithm. Therefore, a critical evaluation of different GFR formulas in the local setting has to be considered, and a combination of several formulas might be useful if a gold standard method is not available.

## Author Contributions

MA, JH, MB, and LW designed the study and were responsible for data collection, analysis, and interpretation. MA and JH drafted the manuscript. AB and LW reviewed the manuscript. All the authors approved the final manuscript.

## Conflict of Interest Statement

The authors declare that the research was conducted in the absence of any commercial or financial relationships that could be construed as a potential conflict of interest.
